# Supplementary material for: An experimental machine learning study investigating the decision-making process of students and qualified radiographers when interpreting radiographic images
Source: PLOS Digit Health. 2023 Oct 25;2(10):e0000229. doi: 10.1371/journal.pdig.0000229 (PMC10599497; doi:10.1371/journal.pdig.0000229)
Supplement: Attachment — Submitted filename: PDIG-D-23-00083.doc [file pdig.0000229.s001.doc]

**Review**

**Manuscript Number:** PDIG-D-23-00083

**Title**: An experimental machine learning study investigating the decision-making process of

students and qualified radiographers when interpreting radiographic images.

**Abstract:** Well written, concise and provides the required information about the study.

- In the following: ‘*Here we report analysis from 106 participants: students (n=?) and qualified radiographers (n=?) as participants of an experimental study’*.
  - Indicate how many students and how many qualified radiographers.

**Introduction:**

- Provides a good overview of the current situation and justification of the study.

**Results:**

- On 1^st^ reference of the area under the curve (AUC), there is no reference to the RoC curve.
  - What was the gold standard used for this RoC study (include in methodology section)?

**Discussion:**

- Good discussion of study findings backed up and compared to literature.

**Materials and Methods:**

- There is a lack of information about the methodology used in conducting the study. In the abstract reference is made to a survey but no details given. In the results, there is reference to interviews. Clarification required.
- What were participants asked to do? Review a set of images?
  - Details pertaining to the above is lacking.
