# Supplementary material for: An experimental machine learning study investigating the decision-making process of students and qualified radiographers when interpreting radiographic images
Source: PLOS Digit Health. 2023 Oct 25;2(10):e0000229. doi: 10.1371/journal.pdig.0000229 (PMC10599497; doi:10.1371/journal.pdig.0000229)
Supplement: Attachment — Submitted filename: Response to reviewers_PlosDH.docx [file pdig.0000229.s002.docx]

**Response to reviewers**

**Received 13^th^ July 2023**

Dear Dr. Neves,

Thank you for the constructive and helpful comments provided by both you and the invited reviewers. These have all been addressed in the newly submitted ‘Revised Manuscript with Track Changes’. A full rebuttal is detailed in the table below.

Thank you again for consideration of this paper.

Kindest regards,

Clare Rainey

|  | **Reviewer comment** | **Authors’ action/Rebuttal** |
| --- | --- | --- |
| Reviewer #1 | ***Abstract***  Here we report analysis from 106 student and qualified radiographers….revise this to indicate how many students and qualified radiographers. | Thank you – addressed now |
|  | A brief regarding the technical operation of the AI tool developed and employed here is critical. | Thank you – detail included in the methods section regarding the AI diagnostic assistance and Boruta random forest |
|  | The language and structure of the abstract could be improved with emphasis on the key implication of the findings to current clinical practice, clear separation between intro/background, methods, results, conclusions. | Thank you – this has been organised as suggested |
|  | ***Introduction***  ….. with promising results reported in many studies. Which studies?? | Thank you – amended |
|  | The introduction seem too long and this could be summarised to present the message in a clearer and succinct form. | Thank you – point taken, and some editing has been undertaken |
|  | Key relevant literatures in the area of previous reports on the workforce under consideration are missing from the introduction and/or discussion: Few that come to mind for your consideration: Malamateniou et al (Radiography); Botwe et al (Radiography); Botwe et al (JMRS); Antwi et al (Insights into Imaging): Malamateniou et al  and Akudjedu et al (all in JMIRS). Some of these are of global relevance and thus, these discussions/perspectives are critical. | Thank you very much – I am familiar with all of these studies and have included them now. |
|  | ***Methods*** Was the study approved by any ethics panel? | This is included now. Apologies for the omission. |
|  | How long was the online survey live for? Any reminders? | Included in methods now – thank you |
|  | How was the survey instrument designed? | Included now. Thank you |
|  | Was Piloting of the tool done and what was the result? | Included now. Thank you |
|  | Pre-test of the survey tool to ensure this was compliant across a range of platforms (e.g.,android vrs iOS, Mac vrs MS, computer vrs mobile etc) | Not conducted explicitly, however, feedback on the face validity of the survey was conducted as part of the pilot study – full detail now included in the Methods section. |
|  | ***Discussion*** The clinical applicability and implications could be discussed a bit more in-depth, this is what will be of interest to readers. | Thank you – additional paragraph added. |
|  | What are the study limitations? |  |
|  | ***General Comments*** Good work – however, please pay attention to grammar and language – at some points there is a mix of both American and British English. | Thank you – this is corrected now. |
| Reviewer #2 | ***Abstract:***  Well written, concise and provides the required information about the study. • In the following: ‘Here we report analysis from 106 participants: students (n=?) and qualified radiographers (n=?) as participants of an experimental study’. >> o Indicate how many students and how many qualified radiographers. | Thank you – included now |
|  | ***Introduction:*** Provides a good overview of the current situation and justification of the study. | Thank you very much |
|  | ***Results:*** On 1st reference of the area under the curve (AUC), there is no reference to the RoC curve.  What was the gold standard used for this RoC study (include in methodology section)? | Thank you, included now, page 14 |
|  | ***Discussion:*** Good discussion of study findings backed up and compared to literature. | Thank you very much |
|  | ***Materials and Methods:*** There is a lack of information about the methodology used in conducting the study. In the abstract reference is made to a survey but no details given. In the results, there is reference to interviews. Clarification required. | Thank you – there have been substantial edits made to the Methods section |
|  | What were participants asked to do? Review a set of images? Details pertaining to the above is lacking. | Thank you – as above |
